# Supplementary material for: Why the COVID-19 pandemic is a traumatic stressor
Source: PLoS One. 2021 Jan 11;16(1):e0240146. doi: 10.1371/journal.pone.0240146 (PMC7799777; doi:10.1371/journal.pone.0240146)
Supplement: Attachment — Submitted filename: Response to Reviewers.docx [file pone.0240146.s001.docx]

**Reviewer #1:**

1. **Within the flow of the introduction, the 3 features mentioned above have been discussed in order, but the cancer sample in P.5 117-1222 seems to be out of line.**

We thank the reviewer for helping us to improve clarity here and have changed the way that the cancer research is introduced in the text (page 6). Specifically, we have clarified how cancer is another example of an event that does not meet the Criterion A definition but is nonetheless associated with PTSD symptomology. Therefore, the cancer sample now fits into the theme of the paragraph, which is focused on the idea of traumatic stress arising only from *exposure to actual or threatened death, serious injury or sexual violation* (i.e., only to certain kinds of serious events).

2. **Although it is understood from the whole of the introduction part that the authors aimed to determine the frequency of covid-related pre/post/peri traumatic stress reactions in the relevant population and to identify the factors that determine the severity of this stress, I recommend adding the primary and secondary objectives or hypothesis of the study clearly and explicitly.**

We have now explicitly labelled the primary and secondary objectives of the study and thank the reviewer for helping us to improve clarity here (pages 6-7).

3. **I understand that 45.2% of the population had a Criterion A event in the past which reminds me that the index population can not be defined as a non-clinic population. It would be better to mention this unforeseeable feature as a limitation in the discussion.**

We thank the reviewer for this comment. But, since research has found that exposure to a Criterion A stressor may be as high as 89.7% in the general population, exposure to these kinds of stressor events may actually be the norm rather than the exception (Kilpatrick, Resnick, Milanak, Miller, Keyes, & Friedman, 2013). Furthermore, just because someone experiences a Criterion A event does not mean they will go on to experience clinical levels of distress/develop Posttraumatic Stress Disorder (Kilpatrick, et al., 2013). Research on clinical populations does not typically classify exposure to a Criterion A stressor as making someone part of a “clinical population.” In any case, we included exposure to a Criterion A event as a variable in our regression so that we were able to examine the impact, if any, of prior trauma exposure on our key dependent variables. For these reasons we have opted not to add a discussion point about our sample being defined as clinical.

4. **The time interval should be specified in terms of having a sense of environmental and social conditions in that countries.**

We agree with the reviewer that this consideration is important—for this reason we explicitly state the time interval of data collection (between April 10-21, 2020; page 8) and have a paragraph at the beginning of our results and discussion section that details the COVID-19 cases in each country at the time of collection, as well as COVID-19 related government directives, and other unrelated psychologically impactful events occurring in this time period (page 11). We also mention the broad time period in the introduction (“in the early weeks of the pandemic”; page 4); all of this information was intended to contextualize the time interval during which we collected the data. We are happy to add any additional social or environmental conditions if the editor has further recommendations.

5. **I wonder if the mean PCL-5 scores were significantly different between those 5 countries. In this study, collecting data from 5 countries at different epidemic stages is an advantage in terms of generalizing the results, however, if the PCL-5 scores differ between countries, the geographical location should be included in the regression model as a variable.**

We thank the reviewer for pointing out this oversight. Further analyses revealed that mean PCL-5 scores did indeed differ by country of residence. Therefore, we have added country of residence as a predictor in our regression analyses (pages 17-19). Our main findings remain unchanged.

6. **A detailed statistical analysis part is missing within the Methods section. Especially the regression method used for predictive modeling should be added.**

We thank the reviewer for helping us to improve the clarity in this section. We have now specified the regression methods we used for each regression when we introduce the results (pages 17-18).

7. **The prevalence of PTSD is 16.5% in direct exposure group whereas this rate is 11.5% in indirect exposure group. The reason for being the majority of those with PTSD positive had indirect exposure is because the rate of indirect exposure in the sample is too high (direct exposure 327, indirect exposure 713). So, it is hard to say subjects with indirect exposure had similar PTSD rates with subjects who had direct exposures.**

We thank the reviewer for highlighting this discrepancy. We have now re-written this section to improve clarity and have highlighted the differences in group size to improve interpretation (page 15): *Overall, of the 13.2% of participants who were classed as PTSD-positive, 60.6% were participants who were not directly exposed to the virus (versus those directly exposed; 39.4%; χ² (1) = .031, φ = .067). However, it should be noted that roughly double the number of participants had been indirectly, rather than directly exposed to the virus. Indeed, when examining within these groups, more participants who had some form of direct virus exposure (n = 327) were PTSD-positive (16.5%), than participants who had no virus exposure (n = 713; PTSD-positive = 11.6%).*

8. **As you mentioned in P.19 414 the anticipated events may reflect generalized anxiety disorder. Do you have any explanatory hypothesis as to how these two concepts differ from each other?**

As this reviewer notes, we do highlight the idea that our results could reflect other mental health disorders such as generalized anxiety disorder. However, we also argue that this possibility is unlikely given: a) the nature of COVID-19 as a *specific* stressful event (PTSD focuses on symptoms that arise from a specific event rather than across a wide variety of situations and circumstances), and b) the nature of our method, which asked participants to anchor their symptoms specifically to the COVID-19 pandemic. We have expanded on these points in the discussion (page 21). Additionally, while we did find evidence for non-specific PTSD-like symptoms related to COVID-19 (e.g., irritability and alertness), we also found evidence for more event-specific symptoms such as intrusive thoughts about future COVID-19 related events (page 15).

**Reviewer #2:**

9. **There are many recent studies that study COVID-19 process as traumatic stress and this manuscript does not mention about them and does not discuss their findings and methodology compared to that studies**

Because this area of research is rapidly changing, with new papers being published daily, our manuscript reflected the available literature at the time we submitted our paper for review (Sept, 2020). We have now conducted another literature search and updated our introduction with relevant papers that have been published since our paper was submitted for review. Specifically, we have included two more citations that show that COVID-19 is associated with exacerbated mental health concerns, including PTSD. In terms of methodological differences, the final paragraph of our introduction highlights the differences between prior research and our current investigation (page 7), and we have expanded this paragraph to include the specific points raised by the reviewer. Specifically, most papers, including the papers published since we submitted our paper for review, simply provide a snapshot of mental health concerns (e.g., PTSD levels) during COVID-19—rather than our more specific aim to explain *why* COVID-19 could be considered a traumatic event. Therefore, other current research is not focused on past and future event exposure, and has not measured symptomology related to past and future events. Relatedly, most past research has not asked participants to index their traumatic stress symptoms to the COVID-19 pandemic. Furthermore, research on COVID-19 as a traumatic stressor has primarily been conducted on Chinese samples—vs. our sample of five western countries.

10. **However, the manuscript is difficult to read and not well organized.**

Please refer to more specific comments 11, 12, and 13 below.

11. **The abstract is difficulty to read and does not present much information about the methodology and results. I suggest that the authors re-write the abstract and describe their methodology and results in more detail.**

We thank the reviewer for helping us to improve the clarity of the abstract. We have modified the abstract by adding more details about the methods and results.

12. **Introduction includes repeated explanations and needs to be tightened up.**

We structured our introduction around three key ideas: that people’s traumatic stress reactions to the COVID-19 pandemic may relate more to: the future than the past; indirect than direct exposure; and stressful events that do not meet Criterion A. We introduce these ideas in the introductory paragraph to frame our aims. We then spend one paragraph on each idea. Within each of these paragraphs we a) state our proposal, b) provide relevant supporting theoretical and empirical evidence, c) summarise how the evidence supports our proposal, and d) apply the proposal to COVID specifically. For example (pages 3-4):

a) “We first turn to the idea of traumatic stress as a problem of the past.”
b) “Addis (9 p. 233) argues that remembering and imagining are “fundamentally the same process” (see also 10)…”
c) “Therefore, traumatic stress symptoms may be a problem of anticipating the future….”
d) “Given the unknown timeline of COVID-19, it seems especially likely that PTSD-like symptoms could arise due to anticipating any number of negative future events…”

The final paragraphs of the introduction summarise our aims and method (pages 6-7).

We have carefully re-examined our introduction for repeated explanations and have taken this reviewer comment as an opportunity for further fine-tuned editing. However, we are reluctant to make more substantive cuts to the introduction, because we believe that doing so would sacrifice clarity. We state our aims in brief up front and then in detail, per the format described above. We acknowledge there are also several parts of the introduction framed along the lines of “but we know PTSD symptoms also arise in X circumstances” but the circumstances are different each time we make one of these statements (i.e., they follow the three key ideas, which are distinct from one another). We also note Reviewer 1’s comment that “This is a well-organized manuscript.” We are happy, however, to take editorial direction on this point.

13. **Results and discussion sections are combined, which makes it difficult to follow. Statistical analysis are also reported in this section. They should be seperated with more specific subheadings.**

We thank the reviewer for this comment, however since, as noted above, Reviewer 1 describes our paper as “well-organized” we respectfully disagree with this stylistic suggestion. Given the nature of our results, we believe that separating the results from the discussion would disrupt fluency and increase repetition—indeed, repetition is another of Reviewer’s 2 concerns. We would also point to our general discussion section (page 20), which summarises all of the main findings in addition to where they are first introduced.

14. **In line 275, it is not clear how authors reach to such a definite conclusion with the previous reported result.**

We thank the reviewer for highlighting the need to improve the clarity of this result and soundness of this conclusion. We have revised the language used here (page 13): *Taken together, our findings support emerging research that COVID-19 is associated with PTSD-like symptomology and other mental health issues.*

15. **Authors do not discuss the current literature on the topic, but just discuss their findings from their own point of view.**

We are unsure which part of the manuscript the reviewer is referring to here, but outside of other relevant research on PTSD (unrelated to COVID-19), we discuss nine recent papers on COVID-19 and mental health in our introduction. Rather than repeating the discussion of this research in our general discussion section, we instead talk about our findings in the context of wider PTSD research pertinent to our main arguments. However, in light of this comment, we have added a paragraph to the discussion that places our findings alongside other COVID-19 research (page 22).

16. **Authors do not discuss the traumatic stress definitions done before the introduction of DSM-5.**

We thank the reviewer for pointing out this oversight in our literature review. We have now discussed some of the major differences between DSM-III, DSM-IV and DSM-5 that pertain to two of our main arguments regarding indirect exposure to traumatic events (page 4-5), and certain kinds of traumatic events (page 6).
